# Ethnomycological study of edible and medicinal mushrooms in Menge District, Asossa Zone, Benshangul Gumuz Region, Ethiopia

**DOI:** 10.1186/s13002-020-00361-9

**Published:** 2020-03-04

**Authors:** Rediet Sitotaw, Ermias Lulekal, Dawit Abate

**Affiliations:** 1 Department of Biology, College of Natural and Computational Sciences, Kotebe Metropolitan University, P.O. Box 31248, Addis Ababa, Ethiopia; 2 Department of Plant Biology and Biodiversity Management, College of Natural Sciences, Addis Ababa University, Addis Ababa, Ethiopia; 3 Department of Microbial, Cellular and Molecular Biology, College of Natural Sciences, Addis Ababa University, P.O. Box 3434, Addis Ababa, Ethiopia

**Keywords:** Conservation, Ethnomycology, Indigenous knowledge, Informants, Wild edible mushrooms

## Abstract

**Background:**

Menge District has long been inhabited by people who have a long tradition of using wild mushrooms mainly as food, source of income, and medicine. Extensive utilization of wild edible mushrooms (WEM) coupled with an ever-increasing population growth, deforestation, and agricultural land expansion threatens fungal diversity and WEM in the area. Hence, this study is aimed at documenting and analyzing the ethnomycological knowledge of the people in order to preserve the dwindling WEM wealth and associated indigenous knowledge.

**Methods:**

Ethnomycological data were collected using semi-structured interviews, focus group discussions, participant observations, and walk-in-the-woods methods. Statistical tests were used to compare the indigenous knowledge and practice of wild mushroom among different informant categories using One-way ANOVA and *t* tests.

**Results:**

A total of 20 ethnomycologically important wild mushroom species belonging to ten genera and six families were identified, of which 15 were reported to be edible in the District. The family *Lyophyllaceae* was represented by the highest number of species (nine species, 45%) followed by *Agaricaceae* (seven species, 35%) and each of the remaining four families had single species representation. Significant difference (*P* < 0.05) was observed on the mean number of WEM reported among different group of respondents. Wild edible mushroom collection habit and practice was significantly (*P* < 0.05) influenced by gender, age, and literacy level. The output of preference ranking exercise indicated *Termitomyces schimperi* was ranked first followed by *Termitomyces letestui*, *Termitomyces microcarpus*, and *Termitomyces eurhizus*as as the most preferred edible mushrooms respectively.

**Conclusion:**

The present study shows that Menge District is rich in wild mushroom diversity and associated indigenous knowledge. However, anthropogenic factors together with loss of indigenous knowledge and very poor conservation efforts threaten the survival of economically and ecologically important mushrooms in the area. Thus, complementary in situ and ex situ mushroom conservation strategy is highly recommended.

## Background

Mushroom hunting refers to the activity of gathering mushrooms in the wild (forests, plantations) and surrounding backyard, farmlands, and grasslands [1] typically for eating. Ethnomycology refers to investigating the many years of man’s interaction and selection and utilization of the most useful mushroom present in the immediate environment [2]. Wild edible mushrooms have been a center of concern among different communities in different parts of the world due to their high-quality protein produced with greater biological efficiency, rich in fiber, minerals, and vitamin content [3]. Moreover, the low fat content, with high proportion of polyunsaturated fatty acids [4] relative to the total content of fatty acids makes them a good source of fat. Besides their use as a food, the ethnomedicinal and ritual use of hallucinogenic mushrooms for divination and curing [2, 5] among traditional peoples in various regions of the world is another important aspect of human-fungi interactions.

Despite the high diversity of wild edible mushroom (WEM) in Africa especially in the tropics [1], the scarcity and lack of ethnomycological reports in many African countries is briefly reviewed. Boa has also shown in his report that countries of the continent where there are better reports regarding WEM utilization includes South Africa, Zambia, Zimbabwe, Nigeria, Congo Democratic Republic Congo, Cameroon, Morocco, and Kenya.

Ethiopia is a multiethnic country and is a home to around 85 different ethnic groups. Mushroom consumption habits and practices of the people in different parts of Ethiopia have not been well documented so far. A growing interest has developed during the last decade in assessing the human-mushrooms interaction among different ethnic groups in Ethiopia. According to a preliminary study by Abate [6], indigenous communities in south and southwestern parts of Ethiopia have a good habit of hunting and consuming wild edible mushrooms from nearby forests during the wet season thus considered mycophlic, while the peoples in the north and northeastern parts are regarded as mycophobic. There are only few ethnomycological reports for studies in the country [4, 7, 8]. Tuno [7] has described mushroom utilization of Mejenger people, an ethnic group who resides in the southwestern part, and [4, 8] reported mushroom consumption habits among people in Kaffa zone. Information on the diversity of mushrooms is still incomplete in the country. For example, in Ethiopia, only *Agaricus campestris*, *Agaricus xanthodermus*, *Agaricus xanthodermulus*, and seven different *Termitomyces* species have been reported [9–11] in southern, central, and northwestern part of the country.

There is a strong emphasis on subsistence uses of wild edible fungi, and their importance to rural people in developing countries, though there are still significant information gaps [1]. This study focuses on traditional knowledge of wild useful mushrooms that has, until now, received little attention in Ethiopia. Assessing and documenting indigenous knowledge and practice of wild edible mushroom utilization in Ethiopia will help to serve as primary information for further research in the field of nutrition and mycomedicine, and to design people-centered natural resource management and biodiversity conservation practices. It is, therefore, imperative to assess and document the knowledge, attitude, practice, and the major barriers to mushroom utilization among peoples in the study area. The output of this study will contribute its part to the knowledge on the WEMs of the country. In addition, it triggers the birth of more similar research outputs pertinent to the complex human mushroom-interaction and the mushroom-based culture of people in different parts of Ethiopia. This scientific investigation on wild mushrooms will also be applied to help to achieve the major development goals, focusing on poverty alleviation and sustainable use of natural resources.

## Methods

### Study area

**Fig. 1:**
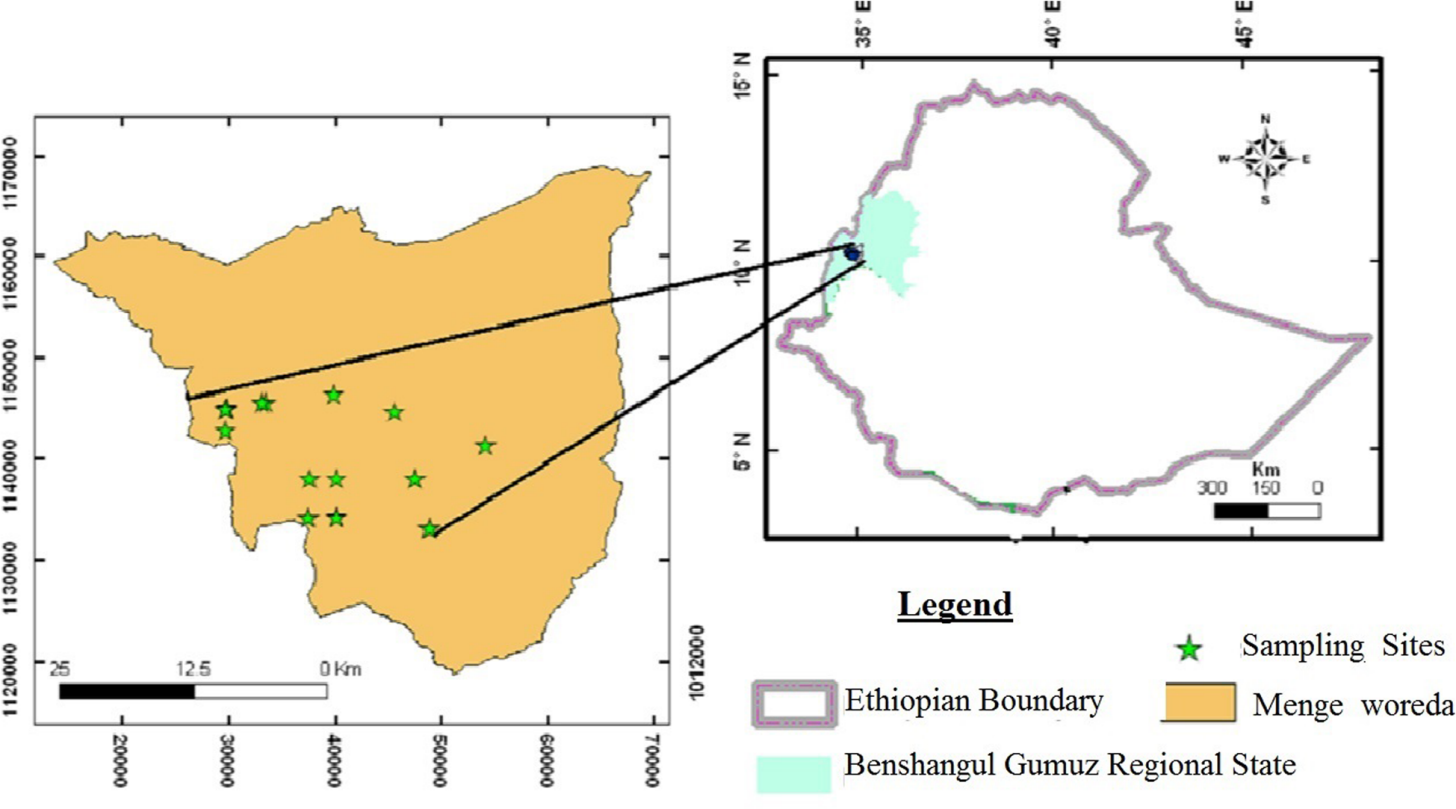
Map of Ethiopia showing the study area

The study was conducted in Menge District located in Asossa zone, Benishangul Gumuz Regional State. The region is located in the northwestern part of Ethiopia (Fig. 1). It is situated in the Blue Nile River Basin and bounded by Amhara, Oromia, Gambella Regional States, and the Republic of Sudan in the north, east, south, and west respectively [12]. Menge District is one of the eight Districts in Asossa zone, which is located 720 km northwest of Addis Ababa and 40 km to the North of Asossa town (the regional capital). It is geographically located between 34° 30′ to 35° 10′ E and 10° 00′ to 10° 30′ N. Topography of the region is composed mainly of the lowland and plains and a few mountainous and gorges, and its altitude ranges from 600 to 1700 m.a.s.l.

### Vegetation of Menge District

About 55% of the total land area of the District is covered with vegetation. The dominant natural vegetation of the district is covered mainly with bamboo thickets (*Oxytenanthera abyssinica* (A.Rich.) Munro), broad-leaved deciduous woodlands dominated by *Anogeissus leiocarpa* (DC.) Guill. & Perr., *Balanites aegyptica* (L.) Delile, *Boswellia papyrifera* (Delile ex Caill.) Hochst., *Combretum collinum* Fresen, *Dalbergia melanoxylon*Guill. & Perr., *Lannea fruticosa* (Hochst. ex A. Rich.) Engl, *Lonchocarpus laxiflorus*Guill. & Perr., *Pterocarpus lucens* Lepr. ex Guill. & Perr., *Piliostigma thonningii* (Schum.) Milne-Redh., *Stereospermum kunthianum*Cham., and *Terminalia laxiflora* Engl [13]**.** and parts of it with grazing and cultivated land. The lowland bamboo tree (*Oxytenanthera abyssinica*) is one of the dominant species in the district covering large areas [12]. The local communities in the district highly depend on forests as sources of construction material, fuel wood, bee keeping, and wild foods. Sorghum, millet, and maize (covering over 70% of the cultivated land) are the most dominant food crops grown in the area. In addition, pulses, vegetables, fruits, ginger, and fiber crops are also commonly cultivated in the district [12].

### Climate of Menge District

Menge District has a mean annual minimum and maximum temperatures of 12 °C and 32 °C, respectively, the lowest occurred in August while the maximum occurred in January. Annual rainfall varies from 800 to 2000 mm. The rainfall in the area is unimodal and obtains high rainfall from May to October where the highest rainfall usually occurs in August. Generally, the rainfall pattern is erratic from year to year [12].

### Ethnographic background and socio-economic aspects

The District has a total population of 40,240 (20,248 males and 19,992 females) and only 1101 (2.7%) are urban inhabitants. The settlement system of the people shows that the rural population is living in remote and inaccessible areas following a scattered settlement system, which are about 12 persons per km^2^ [14]. Recently, the regional government is taking a pivotal measure to help the communities stay within new and permanent resettlements closer into neighborhoods that could have a prime importance in the provision of adequate social services to the people [12, 14].

Majority of the economically active people in the District are engaged in subsistence agriculture including farming, hunting, and forestry. According to [12], only 0.5% of the economically active population is engaged in the industrial sector and 0.7% involved in the service sector. The indigenous people in the region practices shifting hoe cultivation, which is labor intensive. Moreover, the labor-intensive farming tools in the area discouraged people to invest their labor in farming activity. In addition, crop diseases and pests are also serious problems in the region contributing to low crop productivity.

According to the study conducted on the termite challenge in the district, the aggregate annual crop loss in the field and stores from pests was estimated to be about 30–40% [12]. The report has also indicated that people in the region face serious food scarcity from 4 to 6 months annually. As a result, the indigenous people in the region mostly relies on gathering wild foods, hunting, and gold mining to compensate the low production of crops practiced using the traditional shifting hoe cultivation [12]. With regard to ethnic composition, the Berta ethnic group represents the majority of the population in the District. However, there are also other non-indigenous ethnic groups including Amhara and Oromo communities who are the second and the third largest ethnic groups respectively in the area. The Arutana (Berta) language is the most widely spoken language in the area. Afaan Oromo and Amharic are also widely spoken languages in the district. Amharic language is serving as the working language of the study area and the Benishangul Gumuz region as a whole [12, 14].

### Informant selection

The classic methods in ethnobiology were used in the ethnomycological survey of the area. A total of 240 informants (127 male and 113 female) from ten Kebeles (lowest administrative units in Ethiopia) were involved in this study. Representative general and key informants were selected using systematic random and purposive sampling methods following the methods by [8, 15]. Information for nominations of a participant as key respondents was gathered from elderly people and with the help of Kebele administrators. Forty-nine key informants were identified (28 men and 21 women) among the inhabitants. Informed consent was obtained before the start of each interview with general and key informants.

### Data collection

Ethnomycological data and market survey were made in very close interaction with informants using semi-structured interviews, focus group discussions, participant observation, and walk-in-the-woods methods as described in [14]. Face-to-face interviews were conducted in Amharic, Arutana, and/or Oromo language with the assistant of language translators. Socio-demographic profile of the study population, the knowledge, attitude, and practice of human-mushroom interaction which include local names of mushrooms as well as their local uses (medicine, food, etc.), habitat, seasonality of species, marketability, form of mushrooms used (fresh/dried), methods of preparation for food, and preservation (storage) were recorded. In addition information on the source of knowledge, method of indigenous knowledge transfer, the current status/abundance of mushroom, factors affecting the abundance of wild edible mushrooms, and awareness on commercial cultivation of mushrooms were included in the interview. Independent walk-in-the-woods method was employed with key informants for practical identification of wild edible mushrooms in the actual habitat. Participant observations were carried out to study how the indigenous people collect, prepare, and use mushrooms.

Focus group discussions were conducted to gain further information and prove the reliability of the data obtained through individual interviews. Fifteen key informants (eight male and seven female) were involved in the preference ranking exercise as recommended by [15] to identify the most preferred species of wild mushroom for food. Survey in market places of the District was made and the availability, price, who often involves in purchasing, and vending mushrooms was also documented and the extent of use and income generating potential of wild edible mushrooms was analyzed. Dried and/or fresh specimens and colored photographs of representative mushroom specimens were used during interviews and discussions with key respondents and local field assistants. Morphological identification of specimens was performed both in the field and later in the mycology laboratory at Addis Ababa University using taxonomic keys. Authenticated herbarium specimens at Royal Botanic Gardens, Kew, UK and Chinese Academy of Science were also used for identification of specimens. All the specimens were housed at Fungarium, Institute of Microbiology, Chinese Academy of Sciences (HMAS, Herbarium Mycologicum Academiae Sinicae), and duplicates were preserved at Addis Ababa University.

### Data analysis

Descriptive statistics were applied to identify the number and percentage of species, genera, and families of mushrooms used in the community, preferred habitat for mushroom growth, when and how the indigenous people collect wild mushrooms, mechanisms of indigenous knowledge transfer, and conservation practices. Indigenous knowledge dynamics on wild mushrooms use among respondents with different groups, age, sex, family income, and educational level was evaluated statistically using *t* test and one-way ANOVA at 95% confidence level between means using SPSS software version 20. Scores given by key informants on preference-ranking exercise were added and ranked following [16].

## Results

### Diversity of ethnomycologically important wild mushrooms

Although the diversity of species of macrofungi in Menge District is high, the widely shared body of cultural knowledge is restricted to a small group (not more than 20 species of fungi) which belongs to ten genera and six mycological families. The Family Lyophyllaceae was represented by the highest number of species (nine species, 45%) followed by Agaricaceae (seven species, 35%) and each of the remaining four families had single species representation (Table 1).

**Table 1:** List of mushrooms well recognized by peoples in the community, Local names and culinary status and habitat

No	Species/species	Family	Voucher No.	Vernacular name	Use^a^ category			P^b^	Habitat and substrate
					C	M	IC		
1	*Termitomyces striatus* (Beeli) R. Heim	Lyophyllaceae	HMAS273467	Gultse	+	−	−	2	Farm land, on soil
2	*T. eurhizus* (Berk.) R. Heim	Lyophyllaceae	HMAS273459	Tsergunu	+	−	+	2	Forest, termite nests
3	*T. schimperi* (Pat.) R. Heim	Lyophyllaceae	HMAS273460	Zoma/Zip alweta	+	−	+	1	Farm land, termite nests
4	*T. letestui* (Pat.) R. Heim	Lyophyllaceae	HMAS273463	Afifi	+	−	+	1	Farm land, termite nests
5	*T. umkowaani* (Cooke & Massee) D.A. Reid	Lyophyllaceae	HMAS273464	Abenega	+	−	+	2	Grazing land, termite nests
6	*T. robustus* (Beeli) R. Heim	Lyophyllaceae	HMAS273465	Gultse	+	−	+	2	Grazing land, termite nests
7	*T. microcarpus* (Berk. & Broome) R. Heim	Lyophyllaceae	HMAS273461	Aburalu	+	+	−	1	Grazing land, termite nests
8	*T. clypeatus* R. Heim	Lyophyllaceae	HMAS273462	Akukufi	+	+	+	1	Farm land, termite nests
9	*T.* sp.1	Lyophyllaceae	HMAS273467	Angushung	+	−	−	2	Farm land, termite nests
10	*Psathyrella* sp	Psathyrellaceae		Egnegnero	+	−	−	3	Forest,on soil
11	*Laetiporus sulphureus* (Bull.) Murrill	Polyporaceae	HMAS272461	Achechereb	+	+	−	3	Forest, on log
12	*Auricularia* sp*.*	Auriculariaceae	HMAS272463	Huntsul	+	−	−	2	Forest, on living tree
13	*Ganoderma* sp.	Ganodermataceae	HMAS272464	-	−	+	−	−	Farm land, on dead wood
14	*Coprinus comatus* (O.F. Müll.) Pers.	Agaricaceae	HMAS272465	Egnegnero	+	−	−	3	Farm land, on dead wood
15	*Macrolepiota dolichaula (Berk. & Broome) Pegler & R.W. Rayner*	Agaricaceae	HMAS272466	Tsrgunu amigu/ Tsrgunu ashilu	±		−	3	Forest, on leaf litter
16	*M. rhacodes*	Agaricaceae	HMAS272467		±	−	−	3	Forest, on leaf litter
17	*Chlorophyllum molybdites* (G. Mey.) Massee	Agaricaceae	HMAS272468						
18	*Agaricus* sp	Agaricaceae	HMAS272469	Signil tsoro /Gel tsoro	−	−	−	4	Forest, on leaf litter
19	*Leucoagaricus* sp	Agaricaceae	HMAS272470		−	−	−	4	Forest, on leaf litter
20	*Leucocoprinus* sp	Agaricaceae	HMAS272471		−	−	−	4	Forest, on living tree

^**a**^***C*** culinary, ***M*** medicinal, ***IC*** income

^**b**^***P*** palatability (1 = delicious, 2 = good, 3 = just edible, 4 = poisonous)

Ethnomycological findings of this study showed that almost all respondents have a very good knowledge about all the species of termitophilous mushrooms; *Termitomyces clypeatus*, *Termitomyces eurhizus*, *Termitomyces letestui*, *Termitomyces microcarpus*, *Termitomyces schimperi*, *Termitomyces robustus*, *Termitomyces striatus*, *Termitomyces umkowaanii*, and *Termitomyces* sp1 since they are considered to be of excellent quality food by most people. Identification and folk taxonomy of these mushrooms were very easy and consistent across the community. These mushrooms were extensively searched for and were collected excessively than any other mushroom type, either for consumption or for sale.

Generally, 80% of the respondents were found to use mushrooms for food. Among various termitophilous mushrooms, *T*. *microcarpus* and *T*. *clypeatus* were found to serve as medicine by the local people for treating constipation and gastritis in adults and highly recommended for underweight children. The dry powder of *Laetiporus sulphureus* and *Ganoderma* sp*.* were also reported to be used for treating common cold (22%) and wound (18%) respectively. *Coprinus* sp.1 and *Auricularia* sp. were most frequently reported by 40% of informants for their edibility and medicinal uses. Some *Lepiotoid* taxa such as *Macrolepiota dolichaula*, *Macrolepiota procera*, and *Macrolepiota rhacodes* that are commonly known as “tsrgunu amigu” and “tsrgunu ashilu” (referring to mushrooms with annulus on the stipe, and with large umbrella) were also reported for edibility among 13% of the informants.

Five non-edible species were reported for toxicity by the informants. Some species in the genus *Agaricus*, *Chlorophyllum*, *Leucocoprinus*, and *Leucoagaricus* which were growing around animal dung in pastures were generally considered to be inedible, and known by the collective local name “Signil tsoro” and “Gel tsoro” which literally refers to the urine of donkey and dog. All the respondents did not appear to partition this group of macrofungi as finely as termitophilous mushrooms.

According to the respondents, habitat, morphology, color, odor, and test of mushrooms were reported as the common parameters used to distinguish edible from non-edible mushrooms. Beliefs or ideas about the edibility of wild mushrooms available in the study area are summarized below (i–iv):

(i) Wild mushrooms found on termite mounds and rotting woods were generally regarded as edible while mushrooms growing on grazing land and near dung were generally referred inedible.
(ii) Wild mushrooms with mild tastes, thick flesh and eaten by rodents or tortoise were treated as safe for consumption.
(iii) Wild mushrooms changing their color to red, yellowish or blackish after touching or cutting and give burning sensation on tongue when tasted raw were considered to be poisonous.
(iv) Most wild mushrooms with white pilus were cited as poisonous.

### Indigenous knowledge of the community

The average number of WEM reported by each group of informants was compared and higher number of WEM were reported by female respondents; the difference was significant (*P* < 0.05) (Table 2). The number of WEM reported by senior members of the community (> 50 years old) was significantly higher (*P* < 0.05) than young (15–29 years old). There was a significant difference between the average number of mushrooms listed by key informants and general informants. There was no significant difference observed in the number of WEM listed by illiterate and literate informants and among respondents with different family income.

**Table 2:** Statistical test of significance on average number of WEM reported by different informant groups in Menge District

Parameteres	Informant groups	*N*	Average ± SD	*t* value	*P* value
Gender	Female	113	8.13 ± 2.4	7.8	0.006
	Male	127	7.17 ± 2.9		
Age	15–29 (young member)	54	6.31 ± 2.4	10.568	0.00004*
	30–50 (middle age)	101	7.61 ± 2.7		
	> 50 (senior members)	85	8.36 ± 2.5		
Literacy level	Illiterate	168	7.86 ± 2.4	1.512	0.212
	Primary	40	7.15 ± 3.2		
	Secondary	26	6.92 ± 3.1		
	Tertiary	6	7.17 ± 3.6		
Informant category (experience)	Knowledgeable/key	49	10.59 ± 2.3	18.5	0.0000*
	General informants	191	6.86 ± 2.2		
Family income	Low income (< 999)	178	7.72 ± 2.8	0.89	0.412
	Middle income (1000–2000)	52	7.5 ± 2.45		
	High income (> 2000)	10	6.6 ± 2.54		

* Significant difference (*P* < 0.05); *t*(0.05) (two-tailed), df = 238, *N* number of respondents

### Perception, recognition, and folk taxonomy

**Fig. 2:**
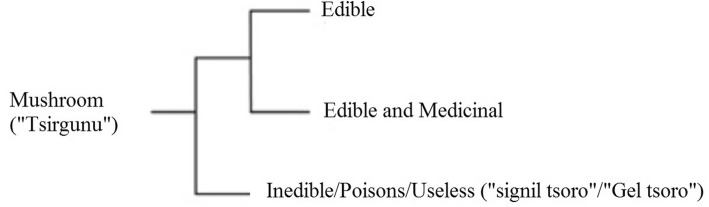
Folk division of domain mushrooms into three smaller groups (Arutana language)

The “total” domain of mushrooms is divided into two general groups among the indigenous community. The first group comprises of a small number (about 20 species) of beneficial and morphologically distinct species about which the community had good ethnoecological knowledge. The second group comprises of a large number (all remaining species) which were grouped together and considered as potentially toxic, useless, or indistinct species about which there is almost no detail knowledge across the respondents (Fig. 2).

All mushrooms were generally known as “Tsergunu” in the District. Specific naming (folk taxonomy) of mushrooms by the Berta communities was mainly based on the substrates where the mushrooms were actually found, the shape of the sporocarp, and growth pattern. For instance, the local name used for *Termitomyces microcarpusis* is “abralu” which means a troop, indicating the typical nature of this mushroom that it occurs in dense crowd, numbering often several thousands. The local term “zip alweta” given to *Termitomyces schimperi* refers to “pinus of the earth” indicating the morphology of the immature sporocarp that looks like a male reproductive organ. The other interesting local name given to all inedible/poisonous/useless mushrooms were “Signil tsoro and “Gel tsoro” which literally means “urine of donkey” and “urine of dog” respectively, which indicate mushrooms that grow in pastureland where cattle were grazing and regarded as non-edible because people believe that the species are contaminated with cattle urine and dung.

The local people’s perception on the origin and development of mushrooms could be grouped into four categories. The first perception was shared by a considerable number (25%) of respondents who believed that mushrooms grow only in the wild without being planted or cultivated. The second groups believed that it was the soil that produces mushrooms. The third perception reflects that it was heavy rain with thunder storm which was responsible for origin and development of mushrooms. The fourth groups represented by majority of the native respondents in the community (60%) reflected that mushrooms grow only by the Divine will and they stated that “Mushrooms are a gift from God to human.”

### Preference ranking

Fifteen key informants were involved in the preference ranking exercise on WEM that were used as food. Termitophilous mushrooms were ranked with top priority. Wood inhabiting mushrooms (*Coprinus comatus* and *Laetiporus sulphureus*) and lepiotoid mushrooms received an average and least ratings respectively in consumer preference as food (Table 3). Mushrooms were mainly reported to be used for food; nevertheless, three additional use categories were also observed in the District. These include the use of mushrooms for medicine, for sale, and as a special gift for respected person.

**Table 3:** Results of simple preference ranking for nine WEM for food

Mushroom species	Informants labeled *R*_1_ to *R*_15_																
	*R*_1_	*R*_2_	*R*_3_	*R*_4_	*R*_5_	*R*_6_	*R*_7_	*R*_8_	*R*_9_	*R*_10_	*R*_11_	*R*_12_	*R*_13_	*R*_14_	*R*_15_	Total	Rank
*Termitomyces striatus*	5	4	3	2	3	6	6	4	3	7	3	5	5	6	4	62	6th
*T. eurhizus*	7	6	5	4	4	9	3	5	7	8	6	7	6	5	6	82	4th
*T. schimperi*	9	8	9	9	7	8	9	9	6	9	9	8	9	8	8	117	1st
*T. letestui*	8	9	7	6	6	7	8	8	9	6	7	9	8	9	9	107	2nd
*T. umkowaanii*	4	5	6	8	8	4	7	7	5	3	5	3	4	5	7	74	5th
*T. microcarpus*	6	7	8	7	9	5	5	6	8	4	8	6	7	7	5	93	3rd
*T. clypeatus*	3	3	4	6	5	2	4	1	4	5	4	4	3	2	3	51	7th
*Coprinus comatus*	2	1	1	3	1	1	1	2	2	2	2	1	2	3	2	23	9th
*T. robustus*	1	2	2	1	2	3	2	3	1	1	1	2	2	1	1	24	8th

Scores in the table indicate ranks given to WEM based on preference as food. Highest number (9) given for the mushroom which informants thought highly preferred as food, (1) is for the least preferred species

### Collection and utilization practice in the District

The main system for obtaining WEM in the community was through direct collection, but in some villages, which were near to the local market, people usually bought it from the market. This study has shown that women in Menge District were highly involved in each stage; collection, marketing, and meal preparation. They deliberately went for mushroom hunting early in the morning on a regular basis since they reported to use WEM both for income and family food consumption during rainy season. However, in most cases, men collect mushrooms unintentionally when they walk to or from their farmland or during hunting.

Results in this study also revealed that there was a relationship between respondent’s sex, age, educational status, and involvement in WEM collection practice (*P* < 0.05). Women were more energy-efficient and gather of a good amount and variety of mushrooms. Men, on the other hand, have a tendency to target some of the most wanted species particularly during food scarcity. Women also tend to collect in groups and usually with their children; in contrast, men were solitary collectors. An adult person spends an average of 3 to 5 h to collect wild mushrooms, and walks about 4 to 10 km for round trip.

There was also a significant difference in WEM collection practice between senior (> 30 years old) and young (15–30 years old) members of the community where elder people (92%) were more involved in WEM collection when compared to younger members (61%) (Table 4). In the case of educational status of the respondents, illiterate members of the community were actively participating in collection than literate people. However, there was no relationship between family income, distance from forest, and involvement in WEM collection.

**Table 4:** Statistical test of significance on percentage of informant groups that involve in WEM collection

Parameters	Informant groups	Involvement in WEM collection?		*P*
		Yes	No	
Sex	Female	107 (94.7%)	6 (5.3%)	0.000**
	Male	88 (69.3%)	39 (30.7%)	
Age	Young (15–30)	33 (61.1%)	21 (38.9%)	0.000**
	Senior (> 30)	172 (92.5%)	14 (7.5%)	
Literacy level	Illiterate	156 (92.9%)	12 (7.1%)	0.000**
	Literate	49 (68.1%)	23 (31.9%)	
Informant category	Key	46 (93%)	3 (6.1%)	0.060
	General	159 (83.2%)	32 (16.8%)	
Family income	Low (< 999)	152 (85.4%)	26 (14.6%)	0.907
	Middle (1000–2000)	44 (84.6%)	8 (15.4%)	
	High (> 2000)	9 (90%)	1 (10%)	
Distance from forest	< 3 km	110 (86.6%)	17 (13.4%)	0.577
	> 3 km	95 (84.1%)	18 (15.9%)	

* Significant difference (*P* < 0.05); ** *t*(0.05) (two-tailed)

There was a significant difference in frequency of collection among different informant groups based on gender, age, literacy level, and experience while there was no significant difference among respondents with different income and the distance of their location from the forest (Table 5).

**Table 5:** Statistical test of significance, on how often the different informant groups involve in WEM collection

Parameters	Informant groups	How often do you collect WEM?			*P* (two-sided)
		Never	Sometimes	Always	
Gender	Female	6 (5.1%)	32 (28.3%)	75 (66.4%)	0.003*
	Male	24 (18.9%)	40 (31.5%)	63 (49.6%)	
Age	Young (15–30)	18 (33.3%)	14 (25.9%)	22 (40.7%)	0.000**
	Senior (> 30)	12 (6.5%)	58 (31.2%)	116 (62.4%)	
Literacy level	Illiterate	10 (6.0%)	50 (29.8%)	108 (64.3%)	0.000**
	Literate	20 (27.8%)	22 (30.6%)	30 (41.7%)	
Informant category	Key	2 (4.1%)	8 (16.3%)	39 (79.6%)	0.002*
	General	28 (14.7%)	64 (33.5%)	99 (51.8%)	
Family income	Low (< 999)	22 (12.4%)	52 (29.2%)	104 (58.4%)	0.960
	Middle (1000–2000)	7 (13.5%)	16 (30.8%)	29 (55.8%)	
	High (> 2000)	1 (10%)	4 (40%)	5 (50%)	
Distance from forest	< 3 km	14 (11%)	46 (36.2%)	67 (52.8%)	0.082
	> 3 km	16 (14.2%)	26 (23%)	71 (62.8%)	

* Significant difference (*P* < 0.05); ** *t*(0.05) (two-tailed)

### Marketability of WEM

During the rainy season, mushrooms were reported to appear as one of the main items in the local markets. Majority of the local people (73%) were involved in mushroom business either as vendors or buyers. During market survey in the district, we found that from the total of 113 female and 127 male respondents, 68 (58%) women and 32 (25%) men reported that they were involved in selling mushrooms.

During each collection trip, a person reported to collect an average of 2 kg of mushroom. Study on local markets in each kebele revealed that price of mushrooms per kg was slightly different depending on the type of mushroom (size, context of sporocarp, test, and delicacy), abundance, and distance of the village from major mushroom collection sites. However, the price range in majority (57%) of the markets were 10–20 Eth birr (0.3–0.6 USD) and in some market (26%) was between 20 and 30 Eth Birr (0.6–1 USD) per kilo gram (1 dollar = 29.0 Eth birr). In some kebeles, 15% of the interviewed people collect mushrooms to sell along the roadside with cheap price (5–10 birr per kg) which was less than half a dollar. Generally, the price for mushroom was higher than most vegetables and lower than that of meat at markets in the District. Mushrooms in the markets were available in both fresh and dried forms. According to the respondents, most people prefer to buy fresh mushrooms (62%) and the rest choose the dried mushroom. All mushrooms sold in the local markets belong to genus *Termitomyces.* The most expensive WEM identified in the market survey was *Termitomyces schimperi* followed by *T*. *letestui*, *T*. *umkowaanii*, and *T*. *eurhizus* as the second, third, and fourth respectively.

### Indigenous knowledge transfer

Mycological knowledge on identification (differentiation of edible from inedible varieties), naming (folk taxonomy), habitat, phenology, and methods of preparation for food and medicine was reported to be transferred by word of mouth. There were no written documents available in the study area pertinent to indigenous knowledge transfer. In addition, the contribution of other sectors such as school, agriculture experts, etc. were also found to be insignificant.

### Phenology

Almost all the respondents reported that the mushroom growing season is strictly associated with the rainy season in the district (Table 6). Both general and key informants agreed that mushrooms fruit from May to September, with July and August being the months with highest abundance for most species. However, according to key informants, the season is slightly variable every year due to the erratic rains in the region. Generally, based on the phenology of the mushrooms, respondents cluster the species into two groups: “mushrooms of the dry season,” in which *Laetiporus sulphureus*, *Auricularia* sp., and *Ganoderma* sp. were included and “mushrooms of the rainy season,” in which the rest of the species were included.

**Table 6:**
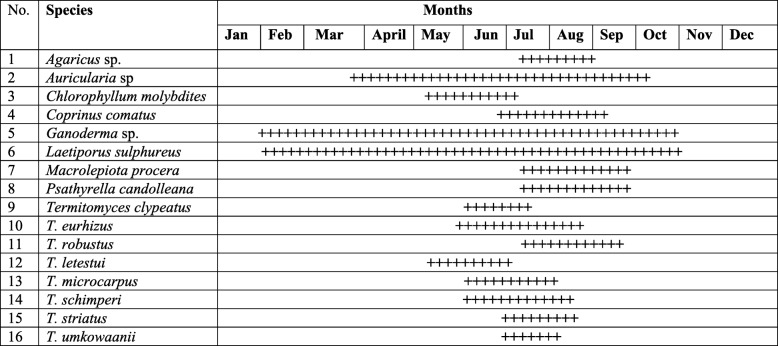
Respondent’s perception about the phenology of different mushrooms type

### Ecology and conservation

**Fig. 3:**
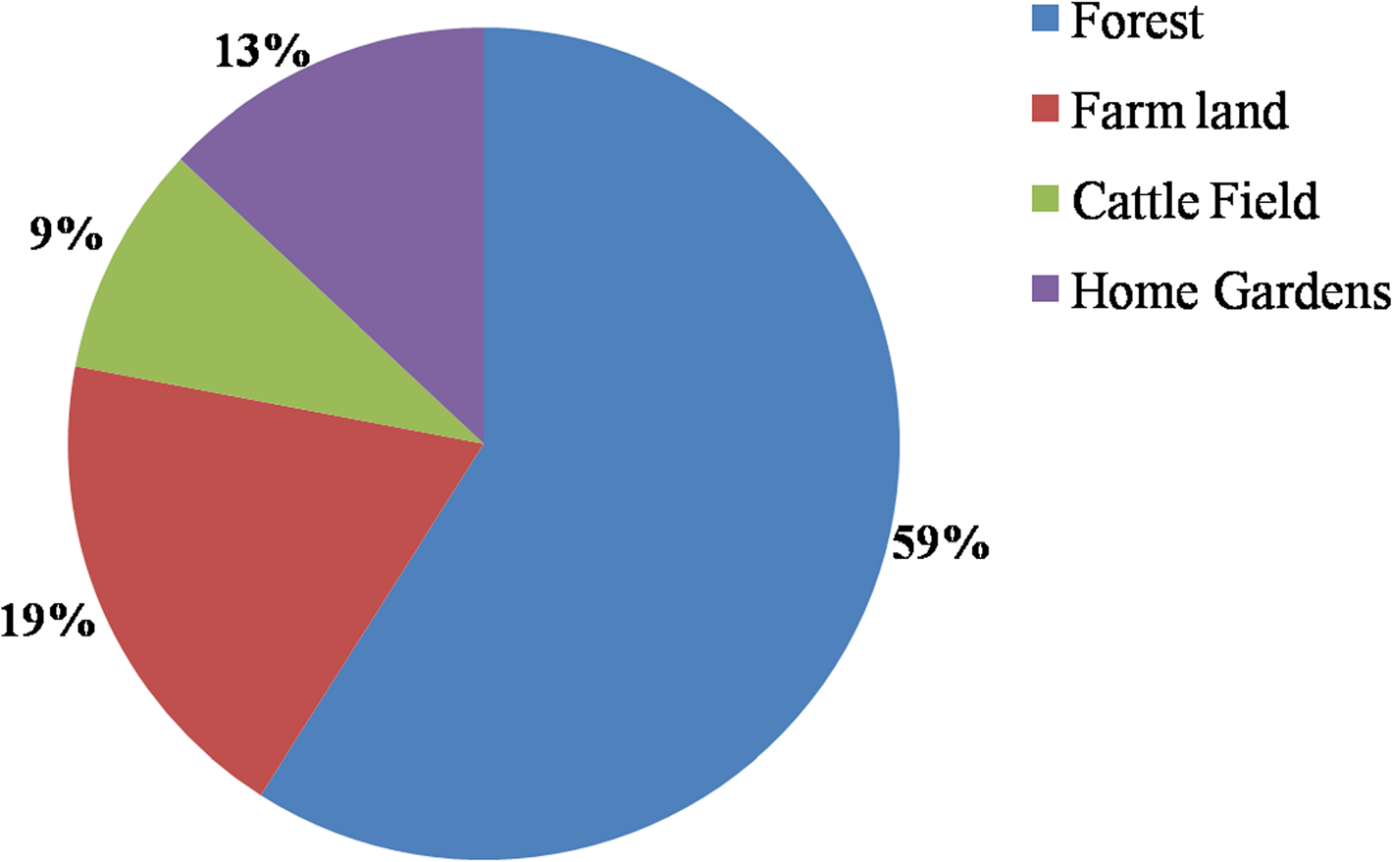
Main habitats with high mushroom distribution recognized by respondents

The indigenous knowledge that people have about mushroom ecology showed variation among individuals in the community and it showed dependence on the activities in which they were engaged as well as their degree of dependence on forest resources. The respondents have recognized three main habitats where the distribution of mushrooms was reported as high. The reported habitats consist of mainly agricultural fields/farm lands, hills and the mountains (harboring forests), and the open areas/plains in most cases where the cattle graze. More than half of the respondents (59%) considered the forest to be the principal area for collecting mushrooms followed by farm lands, home gardens, and cattle fields. The indigenous people showed an extensive degree of knowledge about the places in which diverse mushroom species grow, especially those which were reported to be sold or used regularly (species in the genus *Termitomyces*) (Fig. 3).

The observed poor effort of in-situ conservation of mushrooms together with lack of knowledge on artificial mushroom cultivation, and over harvesting put wild mushrooms under risk of extinction. The major factors affecting wild mushroom wealth of the area as claimed by the respondents were deforestation, agricultural expansion, urbanization/population pressure, and use of agro-chemicals. The respondents > 60% (mostly elderly people) also have indicated that some of the mushrooms they used to consume are no longer found in their locality due to human degradation of the ecosystem through farming activities, use of plants for fire wood and construction, and annual fire outbreak in the environment.

## Discussion

More than hundreds of species of macrofungi were expected to exist in the District; however, the widely shared body of traditional knowledge was limited to a small core group of (about 20) species of fungi. These species tend to represent one of a few common groups: (1) they are either edible/useful or (2) they are morphologically different and not difficult to recognize, (3) considered poisonous, or (4) they are very abundant. Generally, species with unknown toxicity and morphologically challenging to distinct were placed together into a huge group of “useless” species. These species did not have a distinct name/linguistic designation, and generally overlooked. The same type of perception was reported from Mexico and Philippines [16, 17].

Since community members in the study site showed a very good traditional knowledge and practice in the uses of wild edible mushrooms and folk taxonomy, they can be considered as mycophilic. The number of WEM collected and used in the District was found to be higher than reported by [4, 18] in the southwest part of Ethiopia. Results in this study showed the dominance of species from family Lyophyllaceae and Agaricaceae. The extensive use of species from these families might relate to their appealing taste and provision of better income. There was a report from southwest Ethiopia that species in family Lyophyllaceae were the dominant edible mushrooms [4, 18]. This observation is in line with results that have been reported from different parts of Africa and Asia [19–21].

People in the district almost use distinct names to each edible/useful species. This result is also in line with the findings from Tanzania [22], Nigeria [23], and Mexico [16] who had also noted a similar trend of ethnotaxa being comparable to scientific taxa. More than half of the WEM used in the area (53%) were found to be termitophilous mushrooms that belong to the genus *Termitomyces*. This may possibly relate to the fact that the region is located in one of the termite areas and thus the identified species were easily accessible in the nearby areas including home gardens and farmland. The finding agrees with the general pattern of dominance of termitophilous species seen in most WEM studies in the tropical regions [4, 18, 20–22, 24, 25].

Farmlands on which maize and sorghum are cultivated were found to be common areas where most termite comb and the respective *Termitomyces* species were observed. However, the investigation showed that these habitats have been subjected to anthropogenic influences due to the practice of using insecticides to eliminate the termite from the farm. This consequently resulted in decreasing the size of mushroom harvest from such habitat. Misuse of habitats was also observed as the main factor that decreased wild mushrooms distribution and the amount of harvest too [4, 21, 26].

Results also showed the availability of WEM in the local market both in fresh and dried form. However, majority of the respondents (62%) prefer to use it in fresh form while (38%) chose to use the dried mushroom. The regular use of freshly harvested WEM was reported to be associated with the perception of getting better food value, taste, and aroma that they believed could be lost on drying. Mushrooms drying and preservation for later use is a common practice across the world [2, 25, 27].

The average number of WEM reported by different age groups compared in this investigation showed that indigenous knowledge on the use of WEM was significantly higher (*P* < 0.05) among elderly people than in the younger generation which showed knowledge gap between generations. This difference might be due to the influence of urbanization, shift to wage labor, and very poor way of sharing indigenous knowledge through word of mouth. This observation was common not only with WEM but also with other edible and medicinal products collected from the wild [28–31]. Furthermore, the absence of any written document about the cultural use of WEM of the area showed that future use potential of indigenous knowledge is at risk.

Experience in collection and utilization was found to be one of the major variables that influenced traditional knowledge among people in the community. Moreover, the result of this study showed that there is an extremely significant difference (*P* = 0.0001) in traditional knowledge between the key and general informants that might be due to the impact of age-old knowledge and practice in using WEM among the key informants [30, 32]. As described in [1], people with better interaction with wild resource had a better knowledge than those with intermittent interaction.

Ethnomycological studies in Africa have shown that knowledge about mushrooms is extensive, though it varies among countries. However, this knowledge does not indicate a high social valuation or a high consumption of fungi [33, 34]. Even though WEM could be valuable in serving to overcome extreme food shortages in this continent, the amount of harvest in each year have been limited due to anthropogenic and global climate change which have a greater impact in this region of the world [19, 35].

Gender is one of the variables that influence local knowledge distribution. Female informants of the District had reported more WEM on average (8.13 ± 2.4) than male (7.17 ± 2.9) and the difference was statistically significant (*P* = 0.006). As described by Boa (2004), this knowledge difference can be explained at two levels. The first is a consequence of culturally assigned roles for men and women. The second is derived from division of labor because of biological differences between men and women. This result showed that women have better knowledge on use of WEM, since those who were engaged actively in gathering and preparation for food and marketing develop a more profound knowledge on the biology, ecology, and phenology of mushrooms and are able to identify them more specifically [1, 25, 36]. In contrast to ethnobotanical and ethnozoological knowledge, women are typically involved in all the processes of wild edible mushroom utilization [25, 36].

Survey of the major markets in the District indicated that all mushrooms available in the local market were species from the genus *Termitomyces* and the rest of the edible and medicinal mushrooms were not available on market places during the study period. This showed that the genus *Termitomyces* contain species which are highly preferred due to their test, size, nutritional benefit, and ease of access. Among the species sold in the local market, *Termitomyces schimperi* was the most expensive followed by *T*. *letestui*, *T*. *umkowaanii*, and *T*. *eurhizus*. The market value of these species (with a price range from 1 USD to 1.5 USD per approximately 1 kg of fresh mushroom) showed the high demand of these WEM and their contribution to additional income especially for women. The same preference of these mushrooms were reported in most east, west, and central Africa and south East Asia [2, 16, 25, 27, 36, 37]. However, such marketability could also show that the WEM might be over harvested and as a result are under pressure as they are purposefully collected from wild for economic reasons [1, 32].

In most developed countries such as the UK [38], Sweden (where > 50% of the population gathers fungi), Spain, and French [32], it showed that interest of the people in picking and eating WEM has spread throughout the countries. However, different studies have shown mushroom pickers can create a serious disturbance in the forest [32, 38, 39]. Thus in some regions of these countries, some measures have been taken, such as the establishment of legal regulations for the harvest of WEM, the introduction of a license system for mushroom pickers, and distribution of leaflets and posters to raise public awareness about good harvesting practices and the threats of overexploitation [32].

According to the result obtained from the preference ranking exercise, termitophilous mushrooms scored the highest value and considered as the most preferred food among people in the community. In addition, there was a widespread belief that these mushrooms provide essential nutrients, unique flavor, and texture among the culinary tradition of the community. On the other hand, preference of these species may also be related to their relative frequency and abundance in the area. Ethnomycological studies conducted in the south western part of Ethiopia [4, 18] have also reported the common use of species in the genus *Termitomyces.* Medicinal uses of WEM among the community in Menge District is not as strong as similar studies, which have been done in the rest of the world [1, 28, 32]. Most of the inhabitants in Menge District use mushroom mainly as food and rarely for non-food purposes. Only few people (especially key informants) recognized the medicinal contribution of wild mushrooms in the community.

Among useful various mushrooms, *T*. *microcarpus*, *T*. *clypeatus*, *Laetiporus sulphureus*, and *Ganoderma* sp. were found to serve as a medicinal mushrooms. *T*. *microcarpus* was reported for its use against constipation and gastritis in adults while *T*. *clypeatus* were usually given to underweight children. On the other hand, the powders of dried *Ganoderma* sp. and *L*. *sulphureus* were used to speed up wound healing process and to treat common cold respectively. However, medicinal investigation of *L*. *sulphureus* was reported by [40, 41] from the southwest part of Ethiopia (Kaffa Zone) showing its wider use to relieve stomachache and to expel a woman’s retained placenta following delivery. There are also some records about the medicinal property of this mushroom [42, 43].

A significant trend of decreasing the WEM distribution and the amount collected in each mushroom season was witnessed by the residents of the community in the past years. This could mainly be attributed to the decline in forest coverage, expansion of agricultural land, human population pressure, and use of agro-chemicals. The same problem was reported from another part of Ethiopia [4, 18] and many parts of the world especially in the tropical regions [19, 22, 26, 27]. On the other hand, in developed countries such as Europe, North America, Japan, Korea, and Russia, not only the tradition of eating wild edible fungi but also a wise use of these resources is much stronger and appears to have resisted the problem experienced elsewhere [32, 44]. As a result, the harvest of WEM in the aforementioned countries is increasing as compared to the trend seen in the developing countries [1, 32]

Only few (< 10%) residents in the study area have knowledge about artificial cultivation of mushroom. However, majority of the people showed remarkable interest to learn and to involve in mushroom cultivation. Such interest of the people to adopt mushroom cultivation is in line with the investigation made in countries like Tanzania, Cameroon, Nigeria, Japan, and China, in which the people strongly accepted mushrooms cultivation for their obvious nutritional and medicinal values [1, 16, 25, 26]. Moreover, it is in line with the advice of the United Nations Food and Agriculture Organization (FAO) which states that consumption of mushroom guarantees to create a valuable addition to the nutritional quality of the diet of people in developing countries [1, 19]. The cultivation of mushrooms can also serve as a potential source of income to support a family especially women thereby contributing to their economic independence [32].

## Conclusion

Although, people in the district have a good traditional knowledge and practice, the attempt to document, utilize, and conserve these valuable wild resources was very poor. Lack of concern in mushroom research in the district and also in the country has affected negatively the integration of this knowledge as an input in food security and mycomedicinal studies. In addition, the very low concern of supporting and funding mushroom-related research in the country has also contributed to the decline of indigenous knowledge on mushrooms in the area.

With Ethiopian rising population together with an increasing demand for food, domestication and cultivation of indigenous varieties of mushroom can be taken as one of the alternatives to bring food security. Investing on the practice of mushroom cultivation on comparative smallholdings and cheap substrates such as agricultural solid wastes, would surely leads to reduce environmental pollution and creates job opportunities.

Identification of *T*. *microcarpus*, *T*. *clypeatus*, *Laetiporus sulphureus*, and *Ganoderma* sp. as medicinal mushrooms of the district was a sound finding and we recommend that clinical investigations of in vivo or animal studies should be carried out on the medicinal uses of these mushrooms to validate indigenous practices.

It is high time to establish a center of documenting and researching indigenous knowledge of the local people in mushroom cultivation and consumption, besides boosting public awareness and delivery of professional support on WEM utilization. This approach will help not only to bring nutritional security but also it serves to save the widely dwindling mushroom genetic resources in the area. Conservation actions in the district should include protection of the natural forest housing WEM from grazing, human interference, and other anthropogenic influences.

## Data Availability

Please contact author for data requests.
